# Special Notice

**Published:** 1868-02

**Authors:** J. T. Banks

**Affiliations:** Griffin, Ga.


### SPECIAL NOTICE.

All applications for Prize Essays in the Georgia Medical Association, for the next session of this body, are respectfully requested to send their Essays, with *sealed motto*, enclosing the name of author to Dr. J. T. Banks, Chairman of Committee on "Prize Essays," by the 15th of March next.

J. T. BANKS, Griffin, Ga.

Feb. 1868.

